# Enhanced performance of CH_3_NH_3_PbI_3−*x*_Cl_*x*_ perovskite solar cells by CH_3_NH_3_I modification of TiO_2_-perovskite layer interface

**DOI:** 10.1186/s11671-016-1540-4

**Published:** 2016-06-29

**Authors:** Wen Wang, Zongbao Zhang, Yangyang Cai, Jinshan Chen, Jianming Wang, Riyan Huang, Xubing Lu, Xingsen Gao, Lingling Shui, Sujuan Wu, Jun-Ming Liu

**Affiliations:** Institute for Advanced Materials and Laboratory of Quantum Engineering and Quantum Materials, South China Normal University, Guangzhou, 510006 China; South China Academy of Advanced Optoelectronics, South China Normal University, Guangzhou, 510006 China; Laboratory of Solid State Microstructures, Nanjing University, Nanjing, 210093 China

**Keywords:** CH_3_NH_3_I, Interfacial modification, CH_3_NH_3_PbI_3−*x*_Cl_*x*_ perovskite solar cells, Photoelectronic properties, Performance

## Abstract

In this work, perovskite solar cells (PSCs) with CH_3_NH_3_PbI_3-*x*_Cl_*x*_ as active layer and spiro-OMeTAD as hole-transport media have been fabricated by one-step method. The methylammonium iodide (CH_3_NH_3_I) solution with different concentrations is used to modify the interface between mesoporous TiO_2_ (meso-TiO_2_) film and CH_3_NH_3_PbI_3−*x*_Cl_*x*_ perovskite layer. Several techniques including X-ray diffraction, scanning electron microscopy, optical absorption, electrochemical impedance spectroscopy (EIS) and photoluminescence are used to investigate the effect of the interfacial modification. It is found that the interfacial modification by CH_3_NH_3_I enhance the crystallinity and increase the grain size of CH_3_NH_3_PbI_3−*x*_Cl_*x*_ layer, and improve the surface wetting properties of perovskite precursor on meso-TiO_2_ film. The sunlight absorption and external quantum efficiency of PSCs in the visible region with wavelength less than 600 nm have been improved. The Nyquist plots obtained from the EIS suggest that the CH_3_NH_3_I modification can reduce the charge recombination rates. The photoluminescence measurement shows that the exciton dissociation in the modified devices is more effective than that in the control samples. The photovoltaic performance of the modified devices can be significantly improved with respect to the reference (control) devices. The CH_3_NH_3_I modified devices at the optimized concentration demonstrate the average power conversion efficiency of 12.27 % in comparison with the average efficiency of 9.68 % for the reference devices.

**Electronic supplementary material:**

The online version of this article (doi:10.1186/s11671-016-1540-4) contains supplementary material, which is available to authorized users.

## Background

Recently, solar cells based on composites of organometallic halide perovskite have attracted much attention due to their super high absorption coefficients, relatively high carrier mobility and easy fabrication by solution process [1–3]. The efficiency of perovskite (CH_3_NH_3_PbX_3_, *X* = Cl, Br, I)-based photovoltaic devices has greatly increased from 3.8 % to more than 20 % in just a few years [4–6]. It is well known that the microstructure and crystallinity of perovskite layer have important influence on the performance of perovskite solar cells (PSCs) [7]. The morphology of the perovskite films influences on exciton separation, charge transfer, and recombination [8]. The low crystallinity of the perovskite films will result in a strong leakage path and has a negative effect on the charge dynamics of PSCs [5, 9]. However, a precise control of the morphology and crystallinity of perovskite layer remains a critical challenge due to the complex crystal growth mechanism of the perovskite materials. Substantial effort has been done to improve the microstructure of PSCs by adjusting the perovskite crystallization kinetics, such as additives modification [10], composition optimization [11], solvent extraction [12], and controlling the temperature, annealing time, or atmosphere [13–15]. However, a control of the crystalline property and microstructure just by optimizing the fabrication processing seems to be insufficient.

It is known that surface modification has been widely used to improve the performance of organic solar cells and dye-sensitized solar cells [16–19]. Interfacial engineering has been also used as a new strategy to control the morphology of perovskite layer and improve the efficiency of PSCs. It is found that interfacial modification can significantly promote the charge transfer and reduce the recombination rate for those PSCs with metal oxides as electron transport materials [20–22]. It was reported that a modification of the interface between ZnO and perovskite layer using self-assembled monolayer can optimize the morphology of perovskite layer and improve the performance of PSCs [23, 24]. It was also demonstrated that modifying the TiO_2_/CH_3_NH_3_PbI_3_ heterojunction interface by glycine can enhance the photovoltaic performance of two-step solution-processed PSCs [25].

In addition, a modification of the perovskite/TiO_2_ interface with a nanoscale layer of Al_2_O_3_ can reduce the charge losses of the PSCs [26]. Excess CH_3_NH_3_^+^ or methylammonium iodide (CH_3_NH_3_I) is very important for the improvement in the optoelectronic properties of perovskite layer. Better coverage, uniform and pinhole-free perovskite films by adding excess CH_3_NH_3_^+^ to the reactants of perovskite layer can be obtained [27]. During the preparation of perovskite layer by sequential deposition method, a proper addition of CH_3_NH_3_I to PbI_2_ solution not only enhances the absorption but also reduces the recombination rate, resulting in the improvement of efficiency in PSCs [28]. These results suggest that it is promise to introduce CH_3_NH_3_I to modify the interface of PSCs.

Based on these considerations, in this work, the PSCs with the glass/FTO/compact TiO_2_/meso-TiO_2_/CH_3_NH_3_PbI_3−*x*_Cl_*x*_/spiro-OMeTAD/Ag structure are fabricated by the one-step solution method. Here, we choose CH_3_NH_3_I to modify the interface between meso-TiO_2_ and CH_3_NH_3_PbI_3−*x*_Cl_*x*_ perovskite layer and investigate the effect of CH_3_NH_3_I concentration on the microstructure of CH_3_NH_3_PbI_3−*x*_Cl_*x*_ layer and photo-electronic properties of the PSCs. The related mechanism is addressed too. The results show that the CH_3_NH_3_I modification at the optimal concentration can improve the sunlight absorption and external quantum efficiency (EQE) in the visible region at the wavelengths less than 600 nm, reduce the charge recombination rate, and promote the charge transfer, resulting in the enhanced performance. The average power conversion efficiency (PCE) of the PSCs can be enhanced from 9.68 to 12.27 %, respectively.

## Methods

**Fig. 1:**
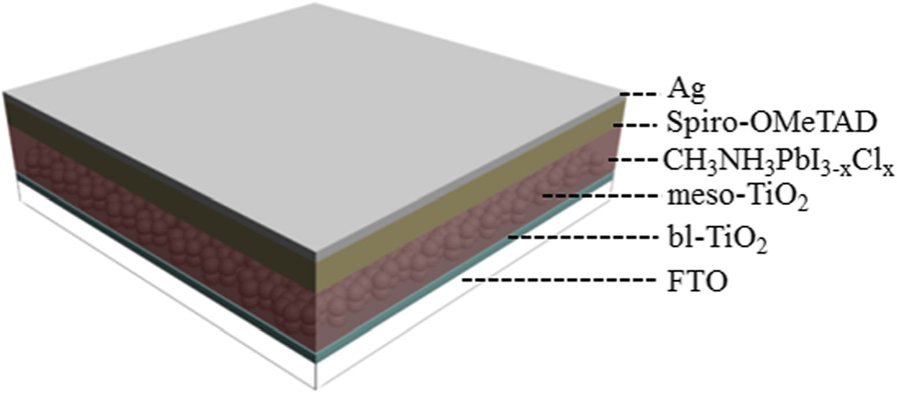
(Color online) A schematic drawing of the perovskite device

Figure 1 shows a schematic diagram of the PSCs fabricated in this work. First, each pre-cleaned FTO substrate was coated with a 60-nm TiO_2_ blocking film by spinning a sol-gel solution (0.25 M titanium isopropoxide in ethanol) at 4000 rpm. The layer was annealed at 500 °C for 30 min to allow sufficient crystallization in ambient air. The meso-TiO_2_ layer was deposited on the TiO_2_ blocking film by spin-coating a TiO_2_ solution (18NR-T, Dyesol) in ethanol at 6000 rpm. These samples were then sintered at 550 °C for 30 min in air to obtain meso-TiO_2_ films. For every batch, several of the as-prepared samples were chosen as the reference samples and the other samples were submitted to next processing.

CH_3_NH_3_I was synthesized using the reported method [3]. For the CH_3_NH_3_I modification, the CH_3_NH_3_I of different concentration dissolved in isopropanol was spin-coated on the meso-TiO_2_ films at 4000 rpm. The untreated samples were chosen as the references. After the modification, these samples together with the reference samples were annealed at 60 °C for 30 min. CH_3_NH_3_I and PbCl_2_ (Aladdin, 99.5 %) were dissolved in *N*,*N*-dimethylformamide (Aladdin, 99.9 %) to obtain a 40 wt % precursor solution with a CH_3_NH_3_I:PbCl_2_ molar ratio of 3:1. The solution was filtered with a 0.45-μm pore size filters before spin-coating. To fabricate the PSCs from the above samples, a CH_3_NH_3_PbI_3−*x*_Cl_*x*_ layer was deposited onto the meso-TiO_2_ film by spin-coating a solution of CH_3_NH_3_PbI_3−*x*_Cl_*x*_ (40 wt % dissolved in DMF) at 2000 rpm for 30 s in the glove box. Then, these samples were annealed in nitrogen (N_2_) ambient at 100 °C for 45 min. Subsequently, 0.08 M spiro-OMeTAD in chlorobenzene solution was spin-coated onto the perovskite film. These samples were left in dry air overnight in the dark. Finally, Ag electrodes with thickness of ~100 nm were evaporated on the sample surface through a shadow mask under a vacuum of 1 × 10^−4^ Pa. All the as-prepared PSCs were fabricated with the standard in-plane size of 3 mm × 4 mm.

### Device Characterizations

The morphology and crystallinity of the perovskite layer were investigated using scanning electron microscopy (SEM, ZEISS ULTRA 55) and the X-ray diffraction (XRD) (X’Pert PRO, Cu Ká radiation). The photovoltaic performance of these PSCs was characterized using a Keithley 2400 source meter under an illumination of 100 mW/cm^2^ (Newport 91160, 150 W solar simulator equipped with an AM 1.5 G filter). The radiation intensity was calibrated by a standard silicon solar cell (certified by NREL) as the reference. The EQE and the UV-vis absorption spectra were measured using a standard EQE system (Newport 66902). The electrochemical impedance spectroscopy (EIS) measurements were performed on the Zahner Zennium electrochemical workstation in the dark. A 20-mV ac-sinusoidal signal source was employed over the constant bias with the frequency ranging from 1 Hz to 4 MHz. The photoluminescence spectra (PL) were measured by a fluorescence spectrophotometer (HITACHI F-5000) exited at 405 nm. The PL spectra have been normalized to the absorbance and measured in the same conditions.

## Results and Discussion

**Fig. 2:**
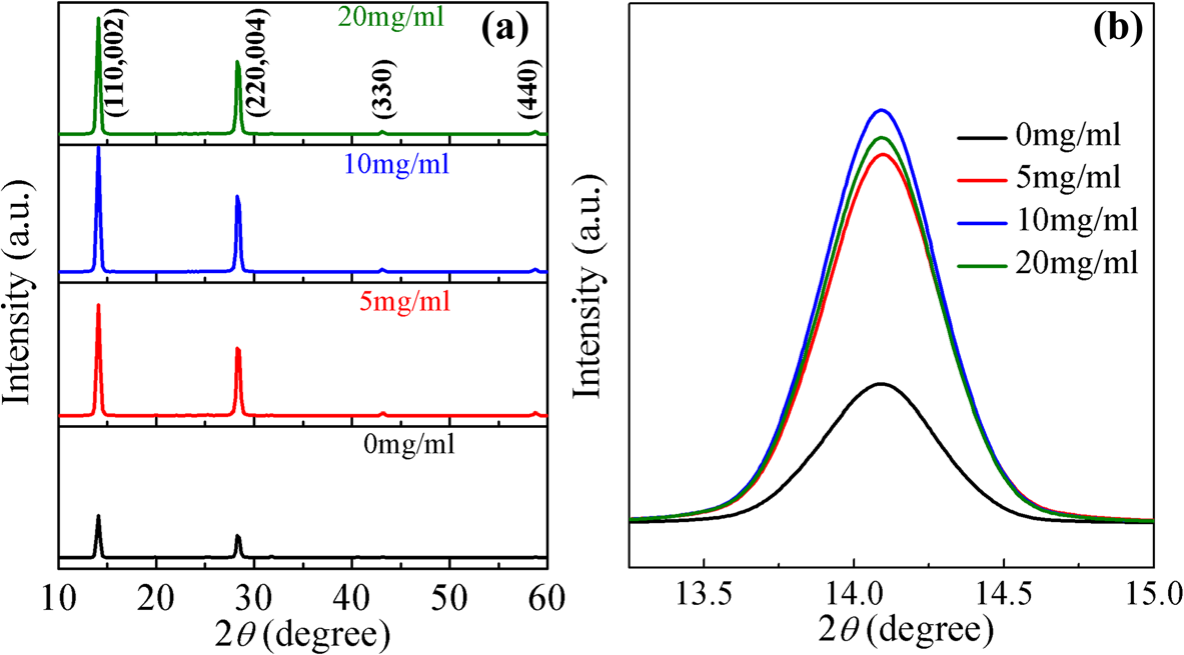
(Color online) **a** X-ray diffraction (XRD) patterns of CH_3_NH_3_PbI_3−*x*_Cl_*x*_ perovskite layer. **b** Detail XRD information of CH_3_NH_3_PbI_3−*x*_Cl_*x*_ from 13° to 15°. The perovskite was deposited on meso-TiO_2_ modified by CH_3_NH_3_I solutions with different concentrations

It is known that the interfacial property has a significant influence on the photovoltaic properties of the PSCs. In this work, it is found that the performance of CH_3_NH_3_PbI_3−*x*_Cl_*x*_ PSCs are influenced remarkably by the concentration of CH_3_NH_3_I solution used to modify the interface between the meso-TiO_2_ and CH_3_NH_3_PbI_3−*x*_Cl_*x*_. To investigate the effect of CH_3_NH_3_I on the performance of PSCs, CH_3_NH_3_I solutions of different concentration at 0, 5, 10, and 20 mg/ml were used, labeled as *x* (*x* = 0, 5, 10, 20). Initially, we investigated the effect of CH_3_NH_3_I modification on the crystalline structure of CH_3_NH_3_PbI_3−*x*_Cl_*x*_ perovskite materials. Figure 2a shows the XRD patterns of CH_3_NH_3_PbI_3−*x*_Cl_*x*_ layers deposited on the meso-TiO_2_ film without and with modification by CH_3_NH_3_I solutions with different concentrations. The peaks at 14.10°, 28.47°, 43.27°, and 58.88° can be attributed to the (110), (220), (330), and (440) reflections of the perovskite crystalline structure, respectively [23]. The presence of these peaks indicates the successful conversion into the perovskite structure, similar to earlier reports [27, 29]. The intensity of all these perovskite diffraction peaks enhances after the CH_3_NH_3_I modification and attains the maximum at *x* = 10. Figure 2b shows the detailed information of the XRD patterns from 13° to 15°. It can be seen that the intensity of (110) characteristic peak increases with the concentration of CH_3_NH_3_I and attains the maximum at *x* = 10 and then decreases with the increase of CH_3_NH_3_I concentration. This implies that the crystallinity of CH_3_NH_3_PbI_3−*x*_Cl_*x*_ film increases upon the CH_3_NH_3_I modification [8]. The improved crystallinity and preferred growth in the (110) direction can be attributed to the excess of CH_3_NH_3_^+^ which slows the crystallization rate of perovskite layer [27, 28].

**Fig. 3:**
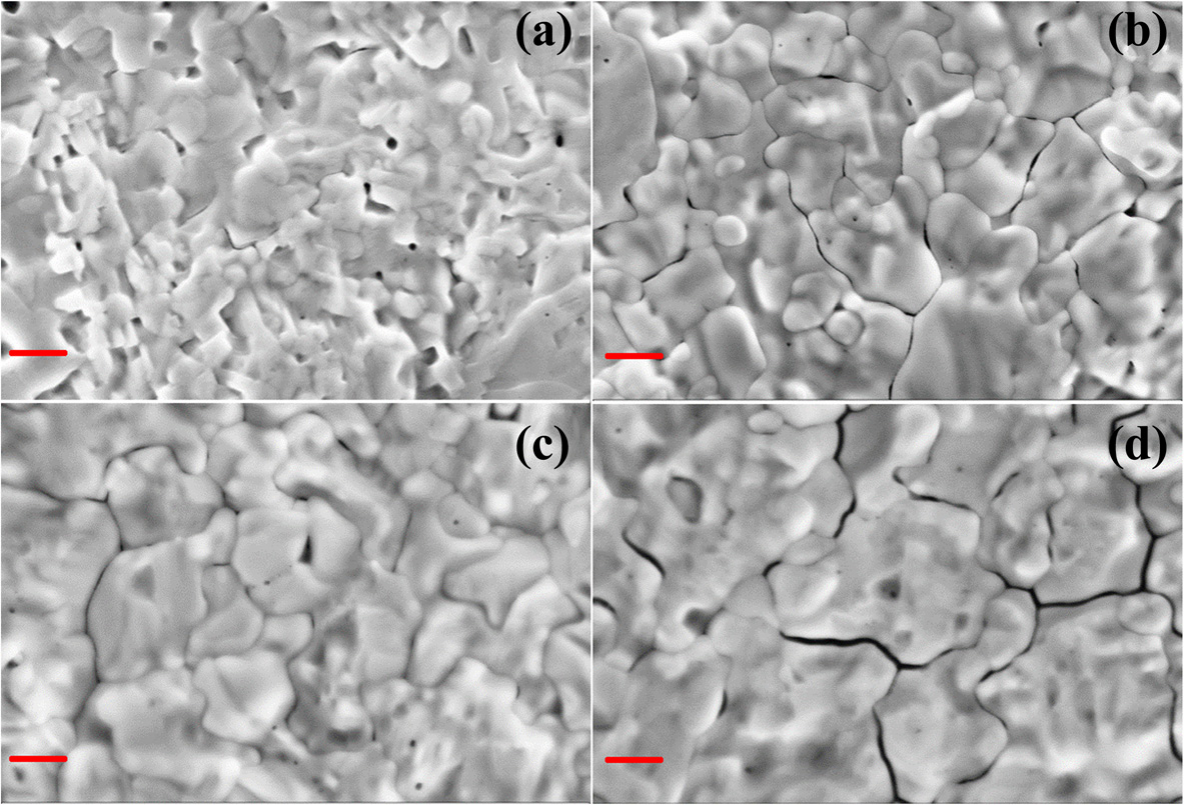
(Color online) Top-view SEM images of the CH_3_NH_3_PbI_3−*x*_Cl_*x*_ films deposited on meso-TiO_2_ films modified by CH_3_NH_3_I solutions with different concentrations **a** 0 mg/ml, **b** 5 mg/ml, **c** 10 mg/ml, and **d** 20 mg/ml, respectively. *Scale bar*, 500 nm

**Fig. 4:**
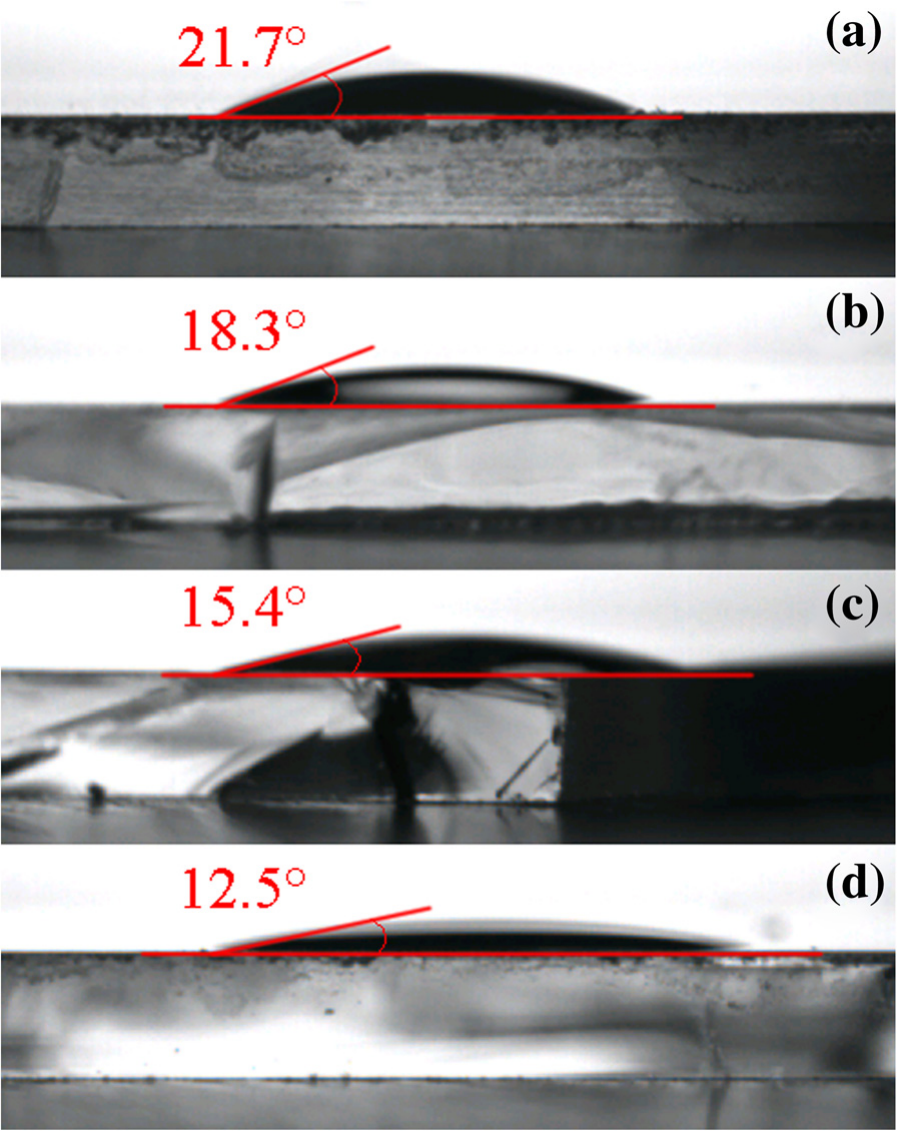
(Color online) Contact angle of perovskite droplet on the meso-TiO_2_ films modified by CH_3_NH_3_I solutions with different concentrations **a** 0 mg/ml, **b** 5 mg/ml, **c** 10 mg/ml, and **d** 20 mg/ml, respectively

The interfacial modification of CH_3_NH_3_I also plays a critical role in the morphology of perovskite layer. The top-view SEM images of CH_3_NH_3_PbI_3−*x*_Cl_*x*_ films deposited on meso-TiO_2_ modified by CH_3_NH_3_I solutions with different concentrations are presented in Fig. 3. It can be seen that the pinholes decrease and the grain size of CH_3_NH_3_PbI_3−*x*_Cl_*x*_ increases upon the CH_3_NH_3_I modification, which will benefit to the performance improvement [9]. For high efficiency PSCs, pinhole-free perovskite films with high crystalline properties are very important. In this view, the enhanced crystalline property and morphology evolution after CH_3_NH_3_I modification may promise an improved device performance of PSCs, which will be discussed below. Figure 4 shows the contact angles of CH_3_NH_3_PbI_3−*x*_Cl_*x*_ precursor solution directly dropped on meso-TiO_2_ with and without CH_3_NH_3_I modification. As seen in Fig. 4, the contact angle decreases with increasing CH_3_NH_3_I concentration. It implies that the surface wetting properties of perovskite precursor on meso-TiO_2_ film are improved after the CH_3_NH_3_I modification, which will facilitate to improve the coverage rates of perovskite layer [30].

**Fig. 5:**
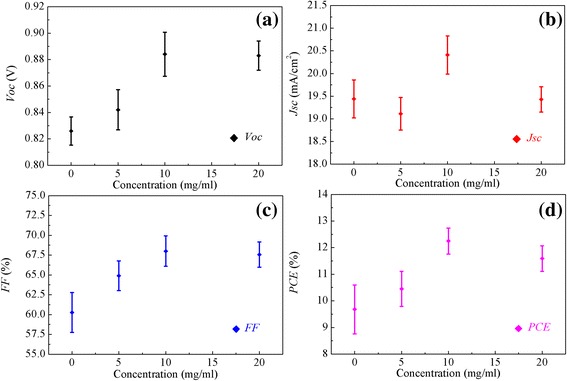
(Color online) **a** *V*_oc_, **b** *J*_sc_, **c** FF, and **d** PCE as a function of CH_3_NH_3_I concentrations for PSCs

To investigate the effect of CH_3_NH_3_I modification on the performance of PSCs, the devices based on the structure illustrated in Fig. 1 are fabricated. Figure 5 shows the detailed photovoltaic parameters including the open-circuit voltage (*V*_oc_), the short-circuit current density (*J*_sc_), fill factors (FF), and PCE for the devices with different CH_3_NH_3_I concentrations. The photovoltaic parameters for those devices are summarized in Table 1. The device without CH_3_NH_3_I modification exhibits an average PCE of 9.68 % and the best PCE of 10.55 %. After the modification by CH_3_NH_3_I solution at *x* = 10, the best PCE of PSCs reaches 12.44 %. The device exhibits *J*_sc_~20.41 mA/cm^2^, *V*_oc_~884 mV, and FF~68.01 %, yielding an average PCE of 12.27 %. The CH_3_NH_3_I modification improves all the device parameters at the optimal concentration of 10 mg/ml. When the concentration of CH_3_NH_3_I is increased to 20 mg/ml, *V*_oc_ and FF decrease, leading to lower PCE. This can be attributed to too much excessive CH_3_NH_3_I caused by the higher concentration, resulting in a redundant impurity to hinder charge transport [27].

**Table 1:** The photovoltaic parameters of the PSCs modified by CH_3_NH_3_I with different concentrations

				PCE (%)	
CH_3_NH_3_I (mg/ml)	*V*_oc_ (mV)	*J*_sc_ (mA/cm^2^)	FF (%)	Averaged	Best
0	826	19.44	60.27	9.68	10.55
5	842	19.11	64.90	10.44	11.21
10	884	20.41	68.01	12.27	12.44
20	883	19.43	67.56	11.59	11.97

**Fig. 6:**
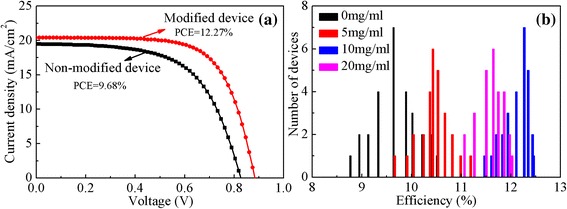
(Color online) **a** The *J*-*V* curves of PSCs without and with CH_3_NH_3_I modification at the optimal concentration (*x* = 10), **b** PCE histogram of PSCs based on meso-TiO_2_ film by CH_3_NH_3_I modification with different concentrations

**Fig. 7:**
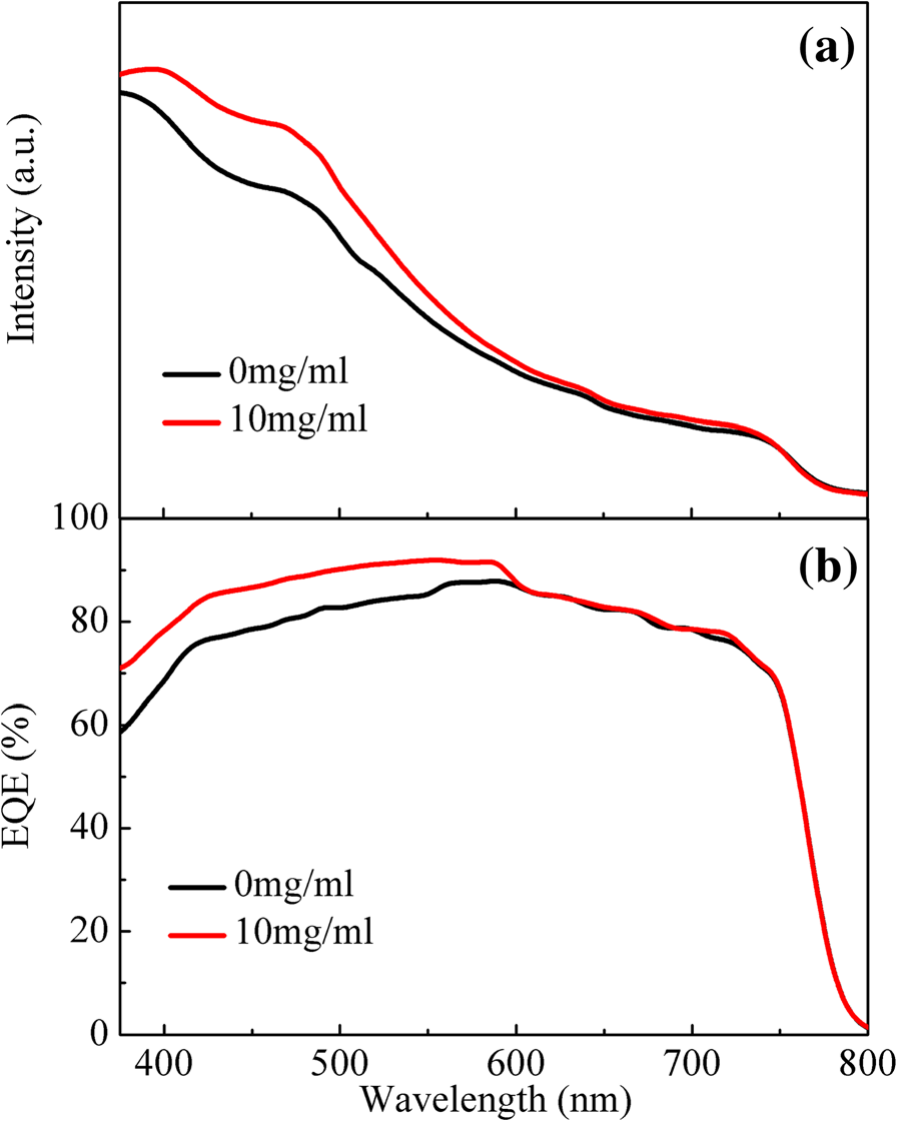
(Color online) **a** Absorbance and **b** external quantum efficiency (EQE) of PSCs without and with CH_3_NH_3_I modification at the optimal concentration (*x* = 10)

Figure 6a presents the *J-V* curves of PSCs without and with CH_3_NH_3_I modification at *x* = 10. Remarkably, the average PCE increases to 12.27 % after CH_3_NH_3_I modification. The introduction of the CH_3_NH_3_I results in significantly enhancement of PCE. The *J*_sc_ increases from 19.44 to 20.41 mA/cm^2^, *V*_oc_ from 826 to 884 mV, FF from 60.3 to 68.0 %, and the average PCE from 9.68 to 12.27 % for the reference device and modified device at the optimal concentration, respectively. For PSCs, the device performance variation is usually observed from batch to batch. In this work, we have fabricated 28 devices for 7 batches to confirm the effect of CH_3_NH_3_I modification on the performance. Figure 6b shows the statistic histogram of PCE for the device without and with the CH_3_NH_3_I modification at different concentrations. The device performance of PSCs with CH_3_NH_3_I modification at the optimal concentration exhibits a narrowed distribution of PCE (range, 11.45 to 12.44 %, with the averaged value of 12.27 %). However, the reference devices show much lower PCE (averaged value 9.68 %) in a wide range (from 8.80 to 10.55 %). Obviously, the improved performance and better reproducibility verified the significance of CH_3_NH_3_I interfacial modification. The possible mechanisms for the enhanced performance of PSCs will be explored below. To further investigate the origin of the increase of *J*_sc_, the absorption spectra and EQE curves for the reference device and modified device by CH_3_NH_3_I solution at the optimal concentration of 10 mg/ml are presented in Fig. 7, respectively. As shown in Fig. 7, the CH_3_NH_3_I modification obviously increases the light absorption and EQE in the visible region at the wavelengths less than 600 nm. The enhanced absorbance and EQE contribute to the improvement of *J*_sc_ in the modified device.

**Fig. 8:**
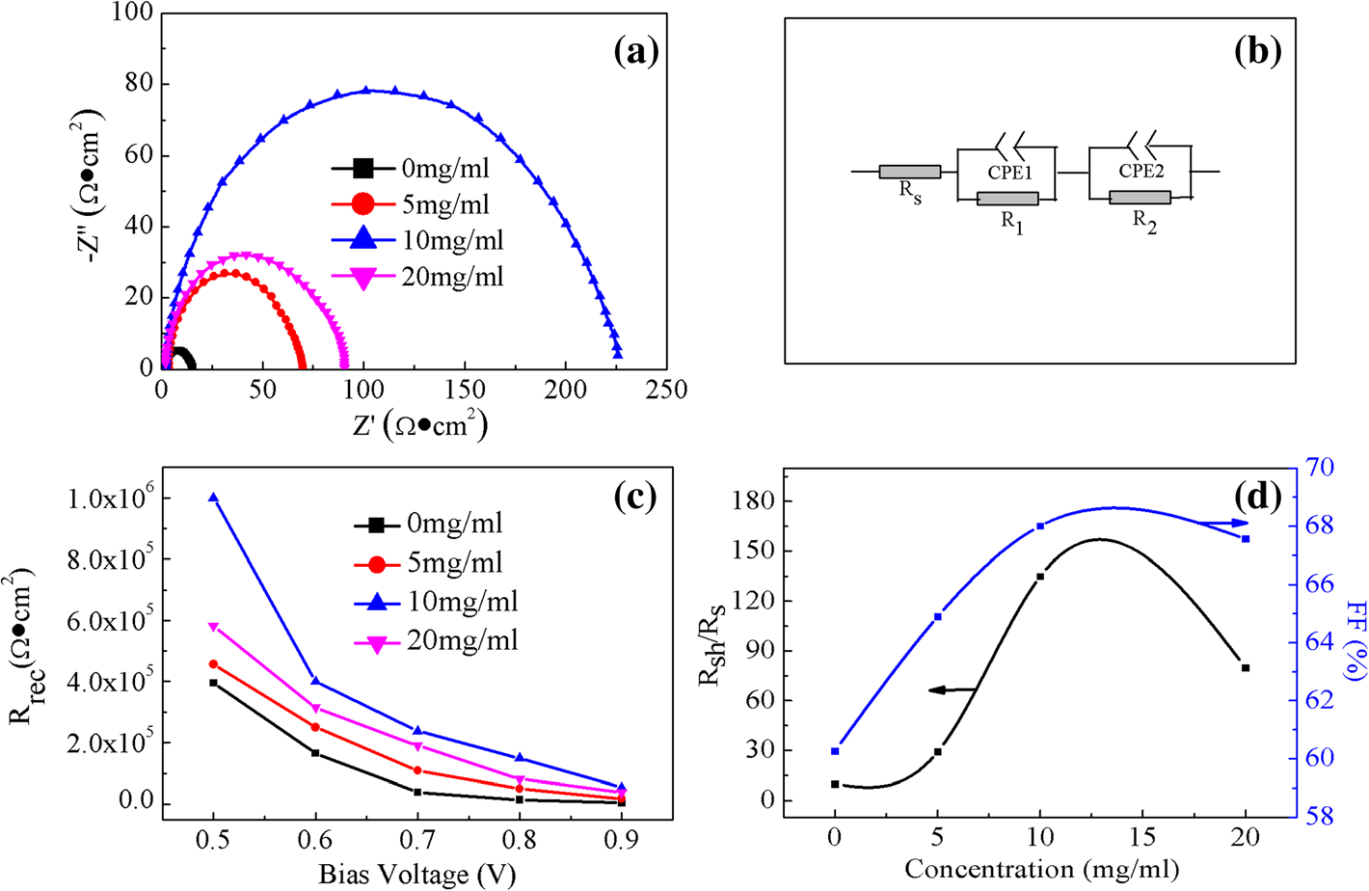
(Color online) **a** Nyquist plots of PSCs modified by CH_3_NH_3_I solution with different concentrations measured at the bias voltage of −0.8 V (close to *V*_oc_) in the dark, **b** Equivalent circuit employed to fit the Nyquist plots. *Solid lines* in **a** are the fittings of the experimental data using the model in panels **b** and **c**. Recombination resistance (*R*_rec_) of PSCs obtained from **b** as a function of concentrations for CH_3_NH_3_I. **d** The ratio of shunt resistance (*R*_sh_) to series resistance (*R*_s_) and FF as a function of concentrations for CH_3_NH_3_I

In order to get a better understanding of the microscopic mechanisms for the observed enhancement of the performance upon the CH_3_NH_3_I modification, the EIS is carried out to characterize the charge transfer dynamics of PSCs. The Nyquist plots for PSCs measured at −0.8 V (close to *V*_oc_) in the dark are presented in Fig. 8a. The solid lines in Fig. 8a are the fits of experimental data using the model in the panel of Fig. 8b. For more accurate fitting, the CPE is used instead of the ideal capacitance C to account for spatial inhomogeneities induced by defects and impurities at the interface. It is can be seen that the measured Nyquist plots can be fitted well by the panel in Fig. 8b. The Nyquist plots consist of two semicircles (See Additional file 1: Figure S1). The first arc at higher frequencies is related to the charge transport and extraction in the Au electrode [30]. The main semicircle is related to the charge recombination at TiO_2_/CH_3_NH_3_PbI_3−*x*_Cl_*x*_/spiro-OMeTAD interface. Similar results have also been reported in the literature [25, 31–34]. The significant difference can be seen in the Nyquist plots of the PSCs with and without CH_3_NH_3_I modification. The size of the arc increases with the increase of the concentration of CH_3_NH_3_I solution and then decreases when the concentration increases to 20 mg/ml, as shown in Fig. 8a. Figure 8c shows the fitted values of the recombination resistance (*R*_rec_) for PSCs without and with CH_3_NH_3_I modification of various concentrations at different bias voltages. It is noted that the device with CH_3_NH_3_I modification exhibits the higher *R*_rec_ than the device without CH_3_NH_3_I modification. It indicates that the recombination rate decreases after the CH_3_NH_3_I modification because the recombination rate is inversely proportional to *R*_rec_ [35]. This will benefit for the charge transfer from perovskite to TiO_2_ [25]. Because all devices are fabricated at the same process except for the CH_3_NH_3_I modification, the difference in recombination rate can be attributed to the interface modification of CH_3_NH_3_I. The device modified by CH_3_NH_3_I solution at *x* = 10 shows the largest *R*_rec_ at the same bias voltage, corresponding to the lowest recombination rate. This result is consistent with the variation tendency of the *V*_oc_ as a function of CH_3_NH_3_I concentrations. It is notable that *V*_oc_ is strongly influenced by the recombination rate at the heterojunction of a solar cell [36, 37]. Lower recombination rate in solar cells will lead to a higher *V*_oc_. Therefore, the significant improvements of the *V*_oc_ and the PCE of the PSCs after CH_3_NH_3_I modification can be understood, which is similar to the effect of surface modification observed in PSCs reported before [23, 31]. Figure 8d shows the plots for the ratio of shunt resistance (*R*_sh_) to series resistance (*R*_s_) and FF for the cells modified by CH_3_NH_3_I solutions with different concentrations. It is reported that the FF depends on the ratio of *R*_sh_ to *R*_s_ [38, 39]. The higher FF value for the cell modified by CH_3_NH_3_I solution is partially attributed to the large ratio of *R*_sh_ to *R*_s_. In short, this PSC modified at the optimal process has the highest *J*_sc_, *V*_oc_, and FF, thus the best performances.

**Fig. 9:**
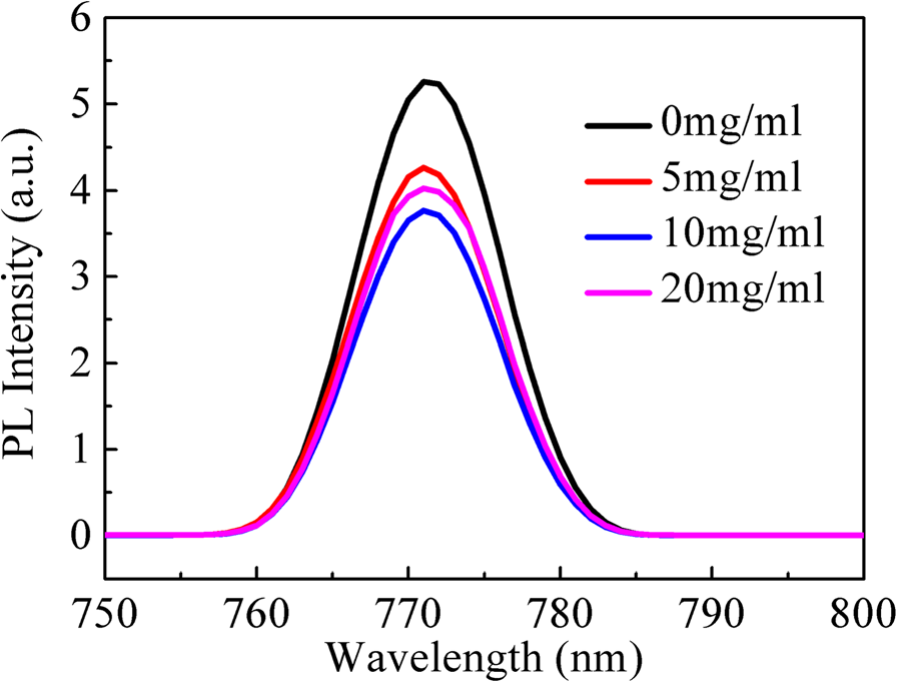
(Color online) Photoluminescence spectra of CH_3_NH_3_PbI_3−*x*_Cl_*x*_ film deposited on meso-TiO_2_ films modified by CH_3_NH_3_I solution with different concentrations

The PL spectra are usually used to explore the trap states and recombination properties of light-excited charge in semiconductors [23, 40–42]. Figure 9 shows the PL spectra of CH_3_NH_3_PbI_3−*x*_Cl_*x*_ films deposited on bare TiO_2_ and modified TiO_2_ by CH_3_NH_3_I solutions with different concentrations. It can be seen that the peak position of the emission is consistent for all of the samples. However, their PL intensities vary a lot and increase with increase of the CH_3_NH_3_I concentration from 0 to 10 mg/ml, then decrease when the concentration increases to 20 mg/ml. The CH_3_NH_3_PbI_3−*x*_Cl_*x*_ film deposited on bare TiO_2_ exhibits the highest intensity in PL spectra, corresponding to a higher charge recombination [23]. The CH_3_NH_3_PbI_3−*x*_Cl_*x*_ film deposited on modified TiO_2_ by CH_3_NH_3_I with the concentration of 10 mg/ml shows the lowest peak intensity, indicating the lowest recombination rate [42] and thus the best photovoltaic performance. This is consistent with the results obtained in EIS characterization (Fig. 8). It confirms that the CH_3_NH_3_I modification on the TiO_2_ layer results in the reduction of recombination rate at the interface between the TiO_2_ and CH_3_NH_3_PbI_3−*x*_Cl_*x*_. The reduced recombination rate of photogenerated charges at the interface can contribute to the enhanced charge collection efficiency in the PSCs, resulting in the improved performance.

## Conclusions

In summary, a series of PSCs based on the structure of glass/FTO/compact TiO_2_/meso-TiO_2_/CH_3_NH_3_PbI_3−*x*_Cl_*x*_/spiro-OMeTAD/Ag have been fabricated. CH_3_NH_3_I are used to modify the interface between meso-TiO_2_ and CH_3_NH_3_PbI_3−*x*_Cl_*x*_. It has been revealed that modifying the interface by CH_3_NH_3_I with appropriate concentration can significantly improve the performance of PSCs. After the CH_3_NH_3_I modification, the PCE of PSCs increases to 12.27 from 9.68 % of the references device. It is suggested that the better performance for CH_3_NH_3_I modified device is mainly attributed to the improved crystalline property, increased sunlight absorption in the visible range and reduced charge recombination rate.

## Additional file

Additional file 1: Figure S1. The Nyquist plots of PSCs modified by CH_3_NH_3_I solution with different concentrations, measured at the bias voltage of −0.8 V (close to *V*_oc_) in the dark. The polts in (a) and (c) correspond to the amplified spectra of (b) and (d) in the high frequency range, respectively. The two R-CPE circuits in series are employed to fit the experimental data in (a) and (b). Only one R-CPE circuit is used to fit the data in (c) and (d). The solid lines are the fittings of the experimental data. It can be seen that the experimental data can be better fitted in Figure S1(a) and S1(b) than that in Figure S1(c) and S1(d). This confirms that the Nyquist plots consist of two semicircles. (TIF 168 kb)
