# Supplementary figures and images for: Enhanced performance of CH3NH3PbI3−xClx perovskite solar cells by CH3NH3I modification of TiO2-perovskite layer interface
Source: Nanoscale Res Lett. 2016 Jun 29;11:316. doi: 10.1186/s11671-016-1540-4 (PMC4927553; doi:10.1186/s11671-016-1540-4)

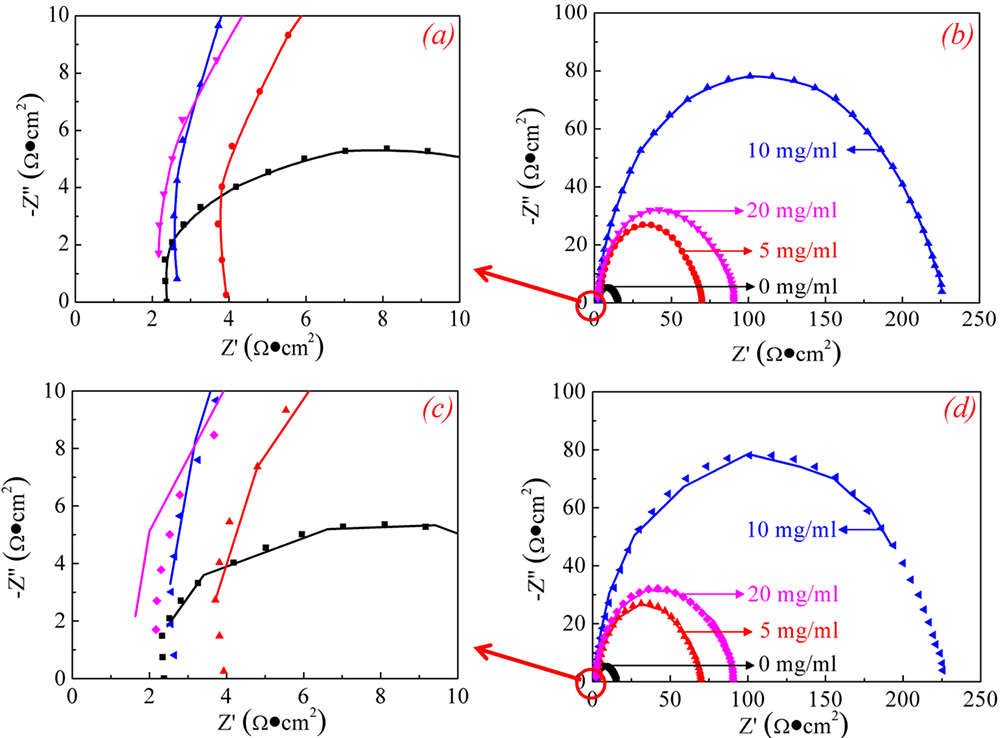

Supplement: Additional file 1: Figure S1. — The Nyquist plots of PSCs modified by CH3NH3I solution with different concentrations, measured at the bias voltage of −0.8 V (close to V oc) in the dark. The polts in (a) and (c) correspond to the amplified spectra of (b) and (d) in the high frequency range, respectively. The two R-CPE circuits in series are employed to fit the experimental data in (a) and (b). Only one R-CPE circuit is used to fit the data in (c) and (d). The solid lines are the fittings of the experimental data. It can be seen that the experimental data can be better fitted in Figure S1(a) and S1(b) than that in Figure S1(c) and S1(d). This confirms that the Nyquist plots consist of two semicircles. (TIF 168 kb) [file 11671_2016_1540_MOESM1_ESM.tif]
